# Supplementary material for: Uncovering hidden bias in neutron diffraction residual strain measurements
Source: J Appl Crystallogr. 2026 Jun 15;59(Pt 4):991–1004. doi: 10.1107/S1600576726004085 (PMC13440052; doi:10.1107/S1600576726004085)
Supplement: Supplementary file 1 [file j-59-00991-sup1.pdf]

JOURNAL OF APPLIED CRYSTALLOGRAPHY

Volume 59 (2026)

Supporting information for article:

Uncovering hidden bias in neutron diffraction residual strain measurements

Cole Franz, Michael Prime, Jeffrey Bunn, Andrew Payzant and Katharine Page

S1. AFSD Deposition Process

Extruded AA6061-T6 bar stock (12.7 x 12.7 mm^2) was deposited onto a rolled AA6061-T6 baseplate (152.4 x 152.4 x 9.5 mm^3) using an L3 discrete feed AFSD machine (MELD Mfg., Christiansburg, VA, USA). In total, 11 unidirectional layers were deposited. The waiting period between each deposition end and start was 1 minute, with 3 minute periods occurring every three layers for feedstock reloading. The tool was constructed primarily of Be-Cu and had an H13 face. On the tool face are four 0.635 mm nub features, and underneath the face is a K-type thermocouple used to control spindle speed through a temperature-controlled feedback loop. During deposition, the spindle speed is constantly adjusted such that the tool thermocouple maintains a setpoint temperature of 425 °C. The process parameters are given in Table S1.

Table S1 AFSD process parameters.

Parameter	Value	Units
Layer Height (h)	2.54	mm
Rotational Speed (ω)	160	rpm
Velocity (V)	3.8	$\frac{mm}{sec}$
Feed Rate (FR)	2.9	$\frac{mm}{sec}$
Temp. Setpoint	420	°C
Tool Nub Height	2.3	mm
Bar Stock	12.7 x 12.7	mm^2

S2. Neutron Diffraction

It is assumed that a macro-scale strain-free reference was achieved by mechanical relief. For this purpose, a 5 mm diameter pillar was sectioned from an identical build, covering the same volumes as those sampled in the un-sectioned component. Photographs of the d_0 texture measurement setup and of the sectioned component are shown in Figure S1.

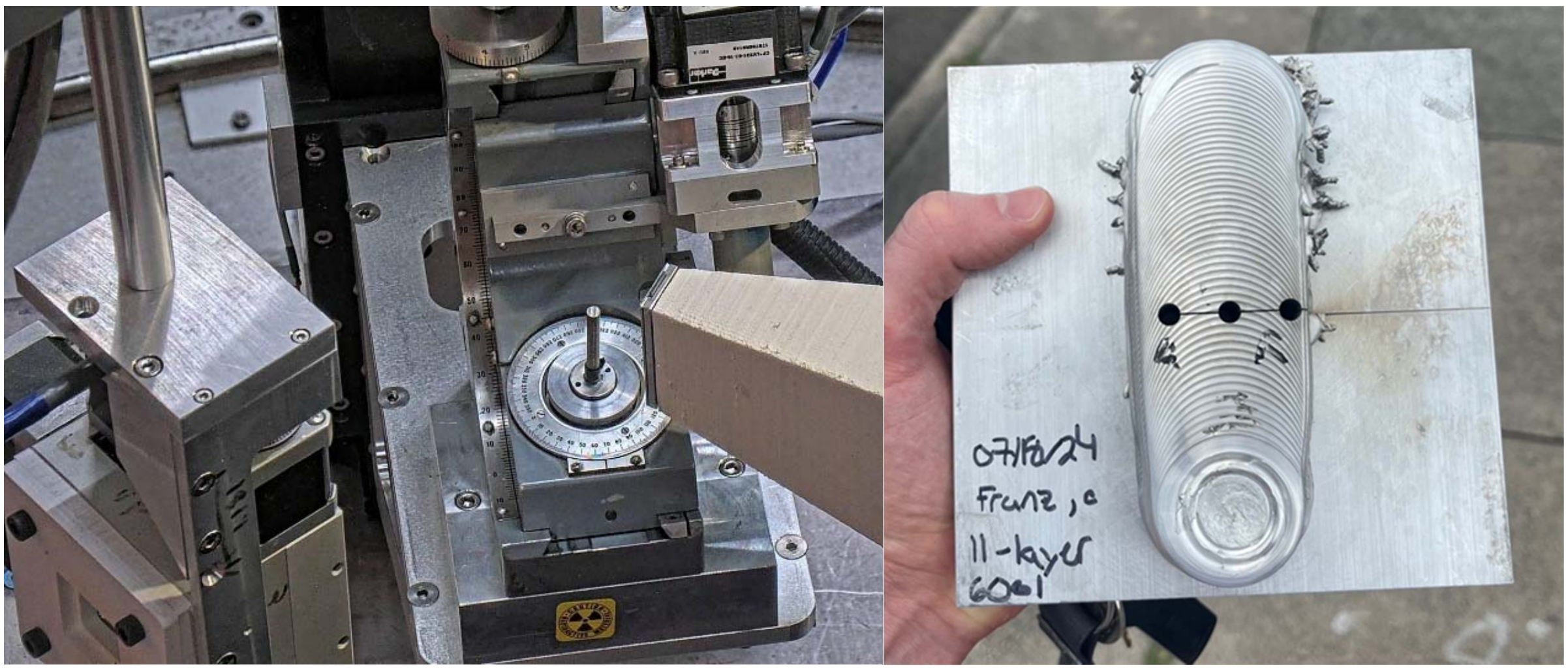

Figure S1 Photographic images of the texture data collection within the d_0 cylinder (left) and of the sectioned component after pillar extraction (right) (only the central pillar was used in this work, following the locations measured in the full component). The bulk stiffness tensor was computed based on single crystal Al properties and the reconstructed orientation distribution functions (ODFs).

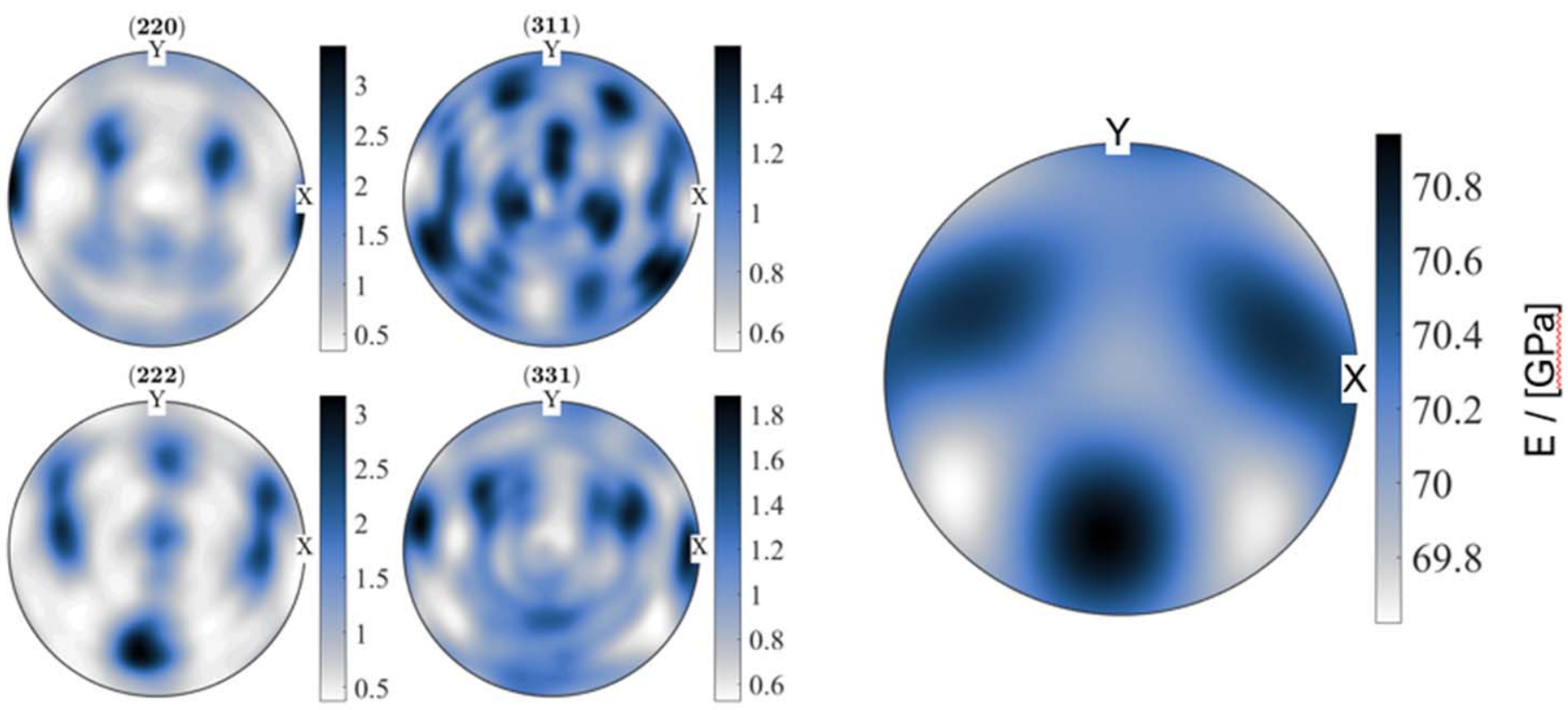

Figure S2 Reconstructed pole figures and bulk-scale elastic modulus based on texture data collected in the top-most volume. No more than 1 GPa variation in stiffness is predicted on the bulk-scale, which warrants use of isotropic elasticity to calculate stresses within the measurement volumes.

S3. Lattice parameter variation

To estimate the theoretical bounds of the unstrained lattice parameter in AA6061, we employed a thermodynamically grounded model based on modifications of Vegard's law with Redlich-Kister Polynomials, which linearly interpolates the lattice parameter from the atomic fractions of constituent elements assuming ideal substitutional mixing:

$$a_0 = \sum_i x_i a_i \quad \text{(S1)}$$

However, in systems like Al-Si-Mg or Al-Mg-Cu where binary interactions are significant, the Redlich-Kister polynomial formulation offers a more accurate description. This includes pairwise interaction terms between solutes *i* and *j*, weighted by their mole fractions:

$$a_0 = \sum_i x_i a_i + \sum_{i=1}^{n} \sum_{j=i+1}^{n} x_i x_j \sum_{n=0}^{k} L_n^{(ij)}(T)\left(x_i - x_j\right)^n \quad \text{(S2)}$$

Here, $L_n^{(ij)}(T)$ are interaction coefficients that depend on temperature. The temperature dependence was included based on coefficients reported by Shin et al. (2017) for Al-Mg, Al-Si, and Al-Cu binary systems (Shin *et al.*, 2017). We implemented these expressions, using temperature-dependent lattice parameters for pure Al, Mg, Si, and Cu and evaluated the resulting lattice parameter of the alloy at room temperature.

To convert this lattice parameter to the expected interplanar spacing for the (311) reflection, we used the cubic lattice relation:

$$d_{311} = \frac{a}{\sqrt{h^2+k^2+l^2}} = \frac{a}{\sqrt{11}} \quad \text{(S3)}$$

Two ternary systems were considered: Al-Si-Mg and Al-Mg-Cu. For each, the full range of solute concentrations permitted by solubility limits or compositional bounds of AA6061 was explored. This included atomic fractions of Mg up to 1.35 at.%, Si up to 0.78 at.%, and Cu up to 0.17 at.%—values representative of the upper limits for supersaturation.

To further refine the model, we considered that some fraction of Mg and Si may be sequestered into Mg_2Si precipitates during solidification or aging. We treated the total alloy composition as a mixture of matrix and precipitate contributions and solved for matrix solute contents $x_{i,m}$,

$$x_{i,\text{total}} = V_p x_{i,p} + \left(1 - V_p\right) x_{i,m} \quad \text{(S4)}$$

where V_p is the precipitate volume fraction and $x_{i,p}$ is the atomic fraction of element *i* in the precipitate. Assuming a stoichiometry of Mg_2Si, we predicted the maximum volume fraction of precipitate that could form before fully depleting one solute. For AA6061 chemistry, Mg was exhausted first, yielding a maximum precipitate volume fraction of 2.01%. Beyond this, any excess Si was assumed to form separate

phases or remain undetected. Based on complete supersaturation and precipitation, the maximum deviation in (311) interplanar spacing is presented in Figure S3.

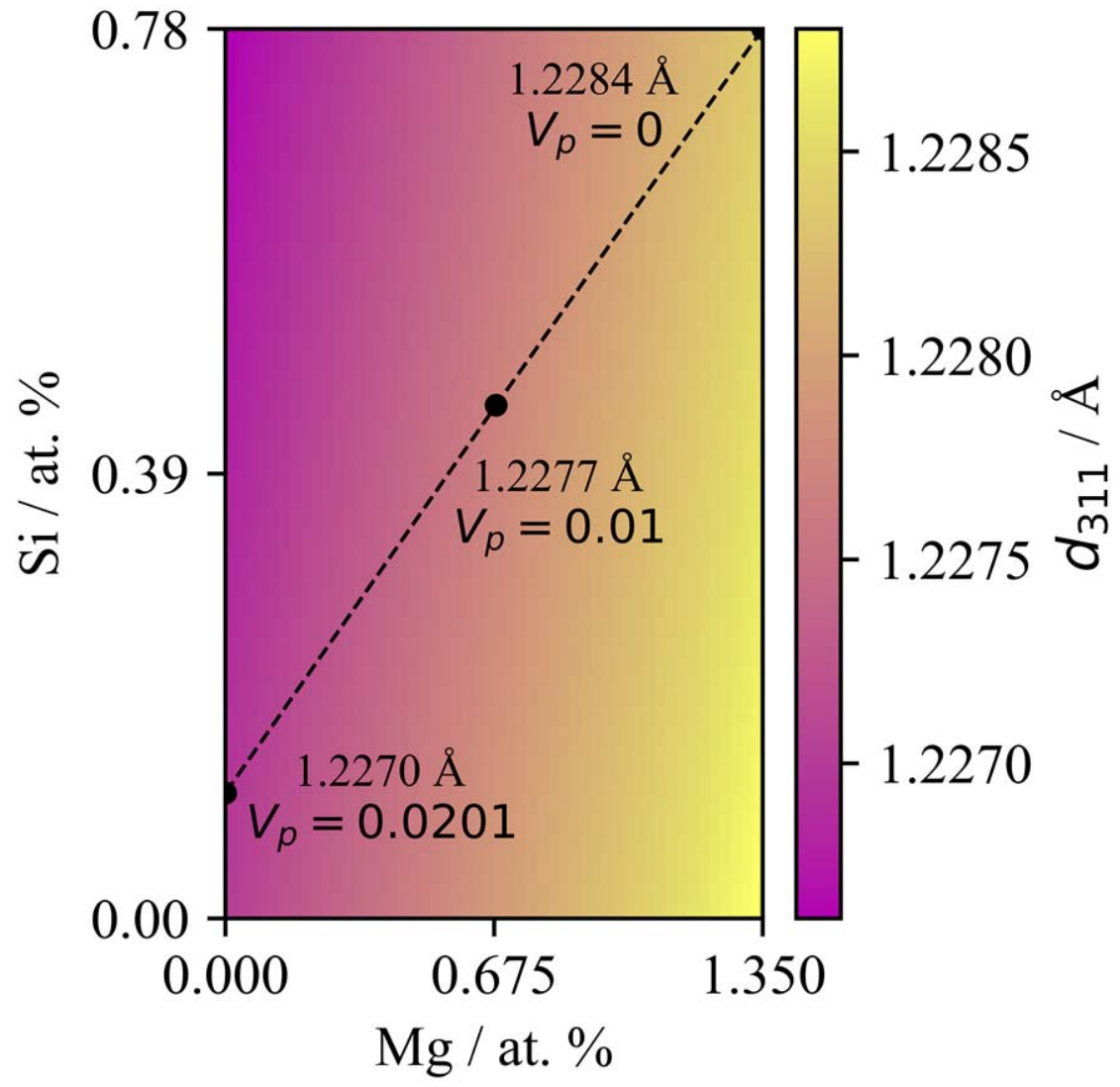

Figure S3 Predicted variation in the (311) interplanar spacing for an Al-Si-Mg alloy system at room temperature. The heatmap represents a range of matrix-level Si and Mg concentrations within the composition limits of AA6061. The dashed line connects three physically relevant points corresponding to different assumed precipitate volume fractions V_p, with their associated d_{311} values annotated. These points represent the maximum solute content in solid solution (no precipitation), partial precipitation (1%), and complete precipitation of Mg (2.01%)—after which no further reduction in matrix Mg is possible.

S4. Six-measurement deviations correlations

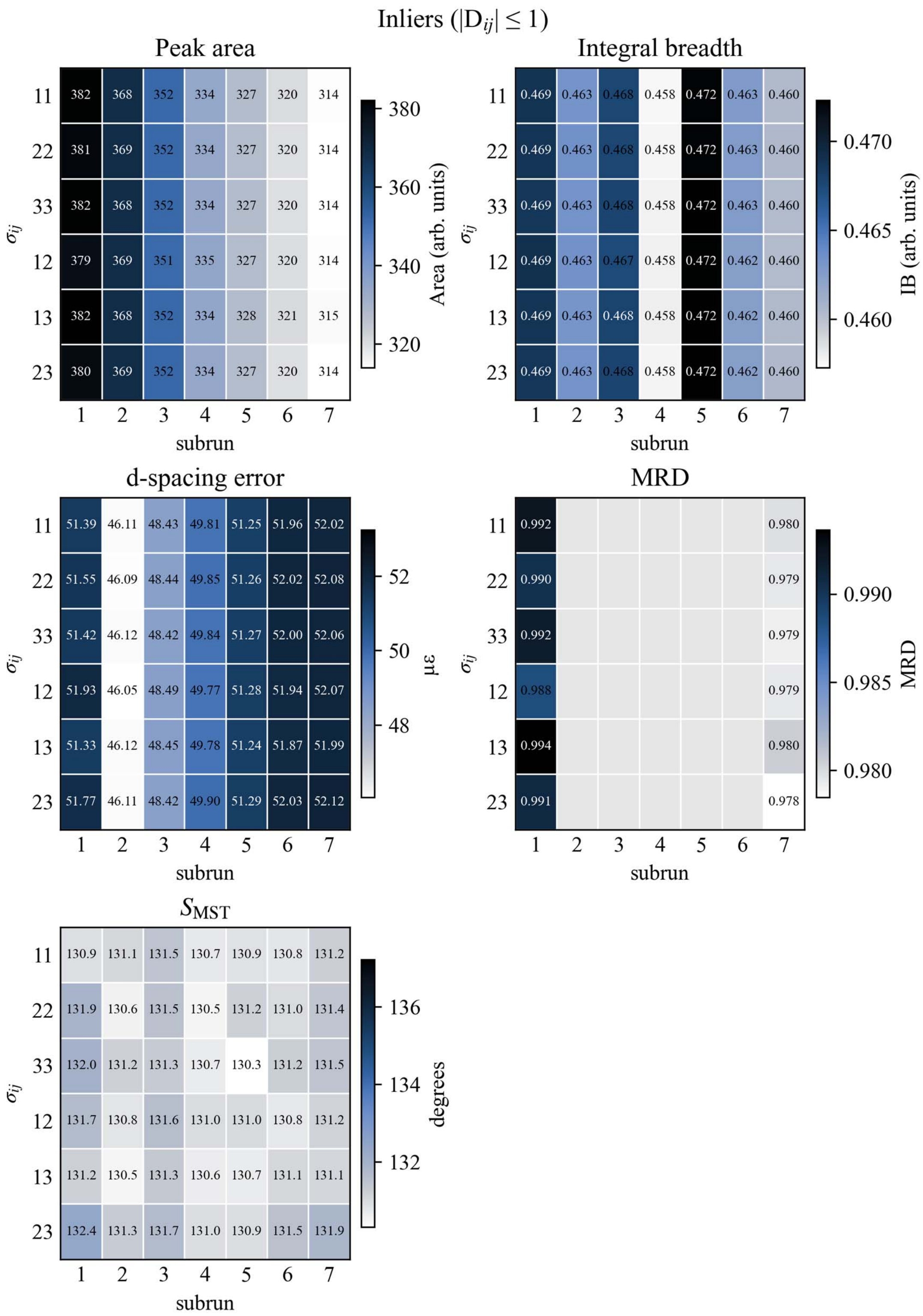

Figure S4 Average metrics of deviation inliers for the six-component solutions to stress.

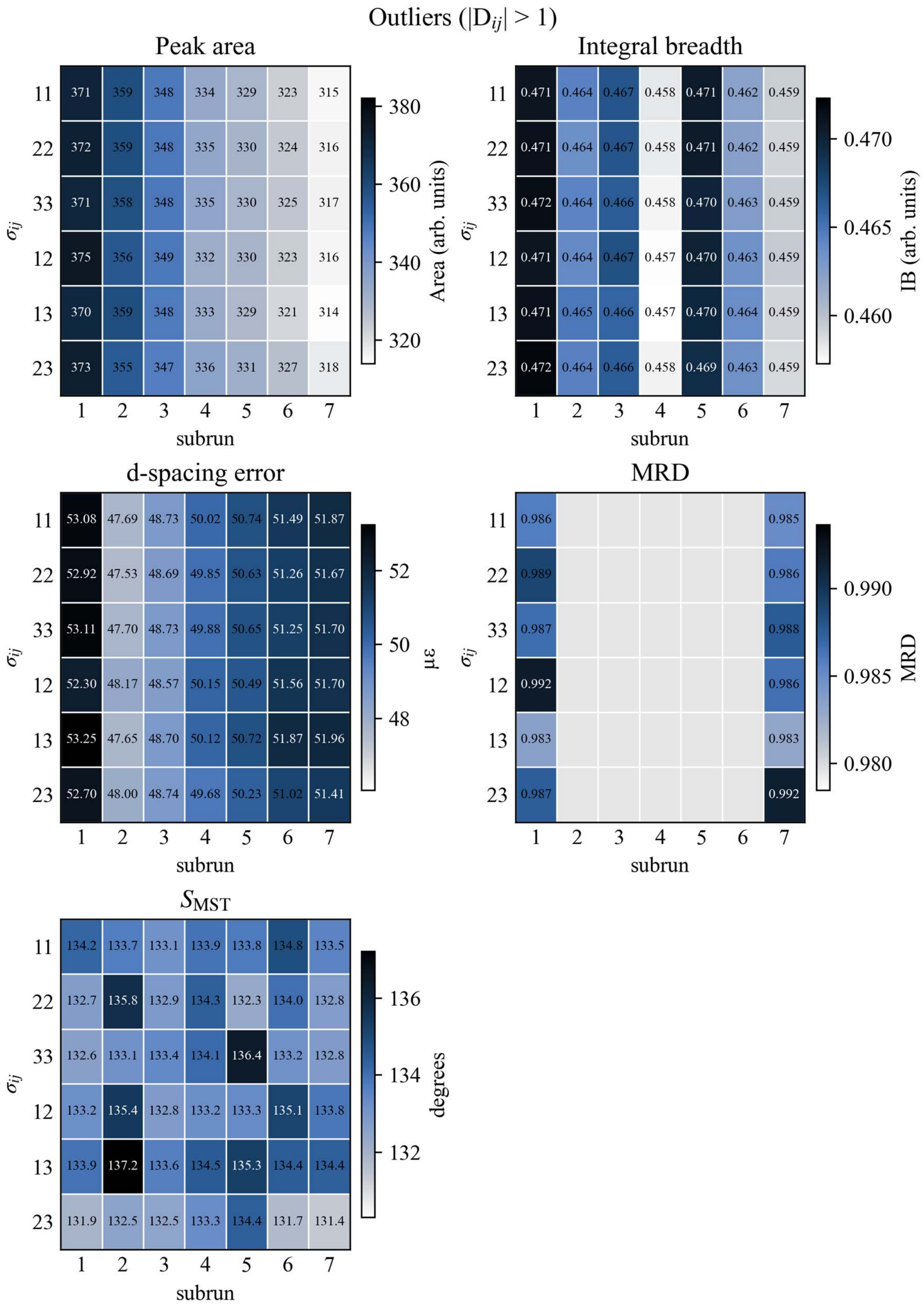

Figure S5 Average metrics of deviation outliers for the six-measurement solutions to stress.

S5. Convergence of least-squares uncertainties

Table S2 Number of orientations of strain measurements within a set for the standard deviation of the nominal solutions and the mean of the error to be less than or equal to ± 100 με.

S_{33} (mm)	ε_{11}	ε_{22}	ε_{33}	ε_{12}	ε_{23}	ε_{13}
-2.8	20	29	28	30	30	25
-5.21	19	29	24	25	27	22
-7.62	20	33	26	24	29	25
-10.16	19	32	26	25	29	25
-12.7	20	32	27	26	29	26
-15.2	21	34	27	27	31	27

S6. Significance of least-squares residuals

To formally assess whether the residuals satisfy the assumptions of zero mean and unit variance, we performed t-tests for bias and χ^2-tests for variance at the 95% confidence level using the weighted residuals computed prior to adding any extra uncertainty. The results for each location are presented in Table S3.

Across all measurement locations, the mean residuals μ_{R_w} were small, and the t-tests showed that none exceeded the critical threshold $t_{0.975,30} = 2.042$. Statistically, this means we cannot reject the null hypothesis that the residual means are zero at the 95% confidence level. However, this does not imply that the strain transformation law is fully valid or that the measurements are free of additional systematic effects. Rather, it indicates that the best-fit tensor lies near the center of the measured data. In contrast, the χ^2-tests for the residual variances rejected the null hypothesis of unit variance at all locations, with χ^2 statistics far exceeding the acceptance interval [17, 47]. This demonstrates that the reported measurement uncertainties alone are insufficient: the residuals exhibit much broader scatter than would be expected

from the nominal peak-fit uncertainties. Physically, this implies that the propagated measurement uncertainties underestimate the true experimental scatter, motivating the introduction of an additional uncertainty scale factor.

A common multiplicative scale factor *C* was therefore applied to the quoted one-standard-deviation measurement uncertainties until the reduced chi-squared of the weighted residuals was equal to unity. The resulting post-calibration residual statistics are reported in Table S4. These values confirm the expected outcome of the calibration procedure and provide the revised uncertainty model used to propagate the least-squares tensor uncertainties.

Table S3: Results of bias and variance significance tests using t-tests, χ2 tests, and 95% confidence intervals for the least-squares estimator without uncertainty inflation (i.e., using only the reported peak-fit uncertainties as the measurement uncertainties).

S_{33} (mm)	μ_{R_w}	σ_{R_w}	t	$t^*_{0.975,30}$	bias test	χ^2_v	$\left[\chi v^2_{0.005,30}, \chi v^2_{0.975,30}\right.$	var. test
-2.8	0.24	2.46	0.59		pass	7.1		fail
-5.2	0.07	1.42	0.28		pass	2.3		fail
-7.6	0.05	1.85	0.15		pass	4.0		fail
-10.2	0.05	1.85	0.17	2.04	pass	4.0	[0.6, 1.6]	fail
-12.7	0.05	1.67	0.19		pass	3.3		fail
-15.2	0.09	1.62	0.32		pass	3.1		fail
-17.8	0.07	1.72	0.26		pass	3.5		fail

Notes: All t-tests assume a 95% confidence interval and degrees of freedom $v = N - p = 36 - 6 = 30$.

Table S4: Results of bias and variance significance tests using t-tests, χ^2 tests, and 95% confidence intervals for the least-squares estimator with uncertainty amplification (i.e., each measurement uncertainty is inflated by constant factor *C*).

S_{33} (mm)	μ_{R_w}	σ_{R_w}	t	$t^*_{0.975,30}$	bias test	χ^2_v	$[\chi^2_{0.005,30}, \chi^2_{0.975,30}]$	var. test
-2.8	0.09	0.9	0.59		pass	1		pass
-5.21	0.04	0.9	0.28		pass	1		pass
-7.62	0.02	0.9	0.15		pass	1		pass
-10.16	0.03	0.9	0.17	2.04	pass	1	[0.6, 1.6]	pass
-12.7	0.03	0.9	0.19		pass	1		pass
-15.24	0.05	0.9	0.32		pass	1		pass
-17.78	0.04	0.9	0.26		pass	1		pass

Notes: All t-tests assume a 95% confidence interval and degrees of freedom $v = N - p = 36 - 6 = 30$.

S7. The effect of counting statistics on uncertainty.

To evaluate whether excess residual scatter is correlated to counting statistics, we computed Spearman's rank correlation coefficient between the absolute least-squares residuals $|R_w|$ and the measured diffraction peak heights for each location. Spearman's ρ measures the strength of a monotonic relationship between two variables as

$$\rho = 1 - \frac{6\sum d_i^2}{n(n^2-1)} \quad \text{(S5)}$$

where d_i is the difference between the ranks of the two variables for the i-th observation, and n is the number of paired observations. Here, $\rho = +1$ indicates a perfect positive monotonic correlation, $\rho = -1$ a perfect negative monotonic correlation, and $\rho = 0$ no monotonic relationship.

For each location, the null hypothesis $H_0: \rho = 0$ assumes no correlation between peak height and residual magnitude, while the alternative hypothesis $H_1: \rho \neq 0$ assumes a correlation exists. To assess statistical significance, ρ was converted to a t-statistic using

$$t = \rho\sqrt{\frac{n-2}{1-\rho^2}} \quad \text{(S6)}$$

which follows a *t*-distribution with $n-2$ degrees of freedom under H_0. The corresponding two-sided p-value was computed as

$$p = 2[1 - F_t(|t|; n-2)] \quad \text{(S7)}$$

where F_t is the cumulative distribution function of the *t*-distribution. Small p-values indicate that a correlation at least as extreme as the observed ρ would be unlikely under H_0, and $p < 0.05$ was taken as evidence to reject H_0 at the 95% confidence level.

Results showed that only location 2 exhibited a statistically significant correlation ($\rho = -0.356$, $p = 0.0328$), indicating a weak negative monotonic relationship between peak height and residual magnitude at this location. All other subruns exhibited $p > 0.05$, implying no statistically significant correlation across most locations. Physically, this suggests that, except for one isolated region, residual magnitude is not systematically influenced by diffraction peak intensity. Thus, counting statistics was not the dominant experimental driver for systematic effects on the strain / stress solutions and uncertainties.
